# Sudanese Medicinal Plants and Their Endophytic Fungi as Sources of Antifungal Agents

**DOI:** 10.3390/plants15203163

**Published:** 2026-10-15

**Authors:** Ali Mahmoud Muddathir, Sakina Yagi, Irmanida Batubara, Johannes Van Staden

**Affiliations:** 1 Research Centre for Plant Growth and Development, School of Agriculture and Science, University of KwaZulu-Natal Pietermaritzburg, Private Bag X01, Scottsville 3209, South Africa; muddathira@ukzn.ac.za or a_muddathir@yahoo.com; 2 Department of Horticulture, Faculty of Agriculture, University of Khartoum, Shambat, Khartoum 13314, Sudan; 3 Department of Botany, Faculty of Science, University of Khartoum, P.O. Box 321, Khartoum 13314, Sudan; sakinayagi@gmail.com; 4 Department of Chemistry, Faculty of Mathematics and Natural Sciences, IPB University, Bogor 16680, Indonesia; ime@apps.ipb.ac.id; 5 Tropical Biopharmaca Research Center, IPB University, Bogor 16128, Indonesia

**Keywords:** Sudanese medicinal plants, antifungal, mycetoma, endophytes

## Abstract

In Sudan, the widespread occurrence of fungal diseases, along with limited access to modern medicine, highlights the therapeutic significance of indigenous medicinal plants. Globally, both medicinal plants and their associated endophytic fungi constitute a major natural reservoir for novel antifungal compounds. This paper reviews the antifungal research undertaken on Sudanese medicinal plants and their endophytic fungi during the period 2000–2025. The literature survey was conducted using the databases Google Scholar, ScienceDirect, PubMed, Scopus, and Web of Science. In this review, a total of 88 plant species belonging to 51 families were investigated for antifungal activity, predominantly against species of *Candida*, *Saccharomyces* and *Aspergillus*. Among these, 16 species were specifically tested against *Madurella mycetomatis*, the primary causative agent of eumycetoma. The family Fabaceae was the most screened family. Analysis of the publications reviewed indicated that the majority of papers used the cup-plate diffusion method for assaying antifungal activity. The minimum inhibitory concentration was the most prominent assay used to detect anti-*M. mycetomatis*. Plant extracts of the Combretaceae family, *Boswellia papyrifera* and *Pimpinella anisum* essential oil, displayed promising antifungal activity. In addition, *Alternaria* sp., *Curvularia* sp. and *Pleospora* sp. endophytic fungi isolated from *Vachellia nilotica* and *Cymbopogon schoenanthus* also showed promising antifungal activity. Further investigation into fungal endophytes is essential to characterize species diversity and identify novel bioactive metabolites. Implementing a modernized research framework for Sudanese medicinal plants is critical for developing novel antifungal leads.

## 1. Introduction

The Republic of Sudan, located in Northeast Africa, is characterized by remarkable ecological diversity ranging from desert and semi-desert regions in the north to tropical savannah ecosystems with seasonal rainfall in the central and southern regions. Sudan features an exceptionally rich flora comprising 3137 species across 1280 genera and 170 families due to its diverse climates, with an estimated 15% of these plants being endemic [1]. Furthermore, this botanical diversity has made Sudan an important reservoir of medicinal plants that have long been used in traditional healthcare systems. Medicinal plants and their secondary metabolites are characterized by diverse chemical structures, low cost, high availability, strong antimicrobial activity, and minimal side effects. Consequently, these metabolites represent promising resources for the identification and development of novel antifungal drugs [2,3]. The traditional medicinal applications of these Sudanese plants have encouraged many pharmacological investigations. Several extracts and purified compounds have been assessed for their biological activities, especially their antibacterial, antifungal, antioxidant, antimalarial, anti-inflammatory, anticancer, and antidiabetic activities [4,5,6,7,8,9,10]. Nevertheless, systematic studies of the efficacy of these extracts and purified compounds on fungal infections remain relatively limited. Despite the therapeutic promise of Sudanese medicinal flora, phytochemical research remains largely limited to crude-extract screening. Advanced fractionation, and compound isolation are comparatively underrepresented in the literature, likely because of restricted access to analytical infrastructure, resource limitations, shortages of specialized personnel and the broader research constraints faced in Sudan [11].

In general, humans are relatively resistant to invasive fungal diseases. However, in immunocompromised patients and HIV-infected patients, the susceptibility toward opportunistic fungal infections is increased. *Candida* spp. are the most common isolates from immunocompromised patients, but *Aspergillus* or *Cryptococcus* may also be isolated from these patients [12,13]. Fungal infections are ubiquitous worldwide; however, their prevalence is more pronounced in sub-Saharan Africa. In Africa, studies indicate that approximately 8% of the Ethiopian population and 11.5% of the Eritrean population get severe fungal infections annually [14,15]. The substantial research on the prevalence of fungal diseases in Sudan revealed that approximately 5 million (10% of the total population) Sudanese individuals are afflicted by fungal infections. The predominant disease was scalp infection (tinea capitis), followed by recurring vulvovaginitis, fungal eye infections, and eumycetoma [16].

Mycetoma, “Madura foot,” is one of the most devastating fungal-associated diseases in Sudan. It is a chronic and destructive inflammatory skin infection caused by aerobic actinomycetes bacteria (actinomycetoma) or fungi (eumycetoma) [17,18,19]. It is classified as a poverty-related neglected tropical disease (NTD), as it predominantly impacts economically disadvantaged communities in endemic areas [20]. Mycetoma is abundant in tropical and subtropical regions, which are known as the mycetoma belt of Africa and encompasses Sudan, Chad, Ethiopia, Somalia, and Senegal [21]. Sudan has one of the highest rates of this disease globally, with more than 16,000 cases reported [16,21]. The primary culprit, *Madurella mycetomatis*, typically infects rural agricultural workers through minor skin wounds [22,23]. Treatment for this infection is notoriously difficult. Standard antifungal medications, which have low cure rates, are highly toxic and need years of adherence; they are also unaffordable for most patients [24]. Frequently, surgical amputation is the only viable option, leaving patients further destitute and disabled [25]. Consequently, there is an urgent need to find novel antifungal medicines that are accessible, safe, and effective.

Natural products continue to provide one of the most productive sources of antimicrobial drug leads, and increasing attention has focused on microorganisms associated with medicinal plants. The term “endophyte” refers to microbes (often fungi and bacteria) that can colonize the inner tissues of healthy plants without the manifestation of any disease symptoms [26]; however, they may become pathogenic when the host plant undergoes senescence [27]. Endophytes associated with medicinal plants can produce complex, highly active secondary metabolites [28]. Some of these microorganisms demonstrate enhanced biosynthetic capacities, likely due to genetic exchange with their host plants while living and proliferating inside healthy tissues [29]. There is some evidence which suggests that endophytic fungi provide a promising source of novel drugs. For example, sordaricin from *Fusarium* spp., are known to possess antifungal capabilities against a variety of infectious disease-causing organisms [30,31]. However, endophytic fungi associated with Sudanese medicinal plants may constitute a promising and largely unexplored source of novel antifungal compounds.

Fungal infections, particularly opportunistic mycoses (*Candida*, *Aspergillus*, and *Cryptococcus*) and NTD such as mycetoma, present a growing global health threat intensified by drug resistance and therapeutic limitations. Although Sudanese medicinal flora and associated fungal endophytes offer rich potential for novel drug discovery, existing research remains fragmented by inconsistent extraction protocols and unstandardized bioassays. The primary objectives of this review are to synthesize literature published between 2000 and 2025, consolidate scattered data on high-promise taxa, evaluate endophytes as sustainable alternatives to wild harvest, and identify current methodological bottlenecks in antifungal research. Ultimately, we highlight key priorities and methodological frameworks to assist future studies in antifungal drug discovery.

## 2. Methodology

Strategy for searching the literature: A literature review was conducted utilizing various electronic databases, including Google Scholar, ScienceDirect, PubMed, Scopus, and Web of Science. The search strategy was conducted using specific combinations of keywords and Boolean operators. The main search strings applied across databases included:

- (“antifungal” OR “antimicrobial “) AND (“Sudan” OR “Sudanese”) AND “medicinal plants”
- “mycetoma” AND (“Sudan” OR “Sudanese”) AND “medicinal plants”
- “endophytes” AND (“Sudan” OR “Sudanese”) AND “medicinal plants”
- These core search combinations were adapted as required for each database.

**Figure 1:**
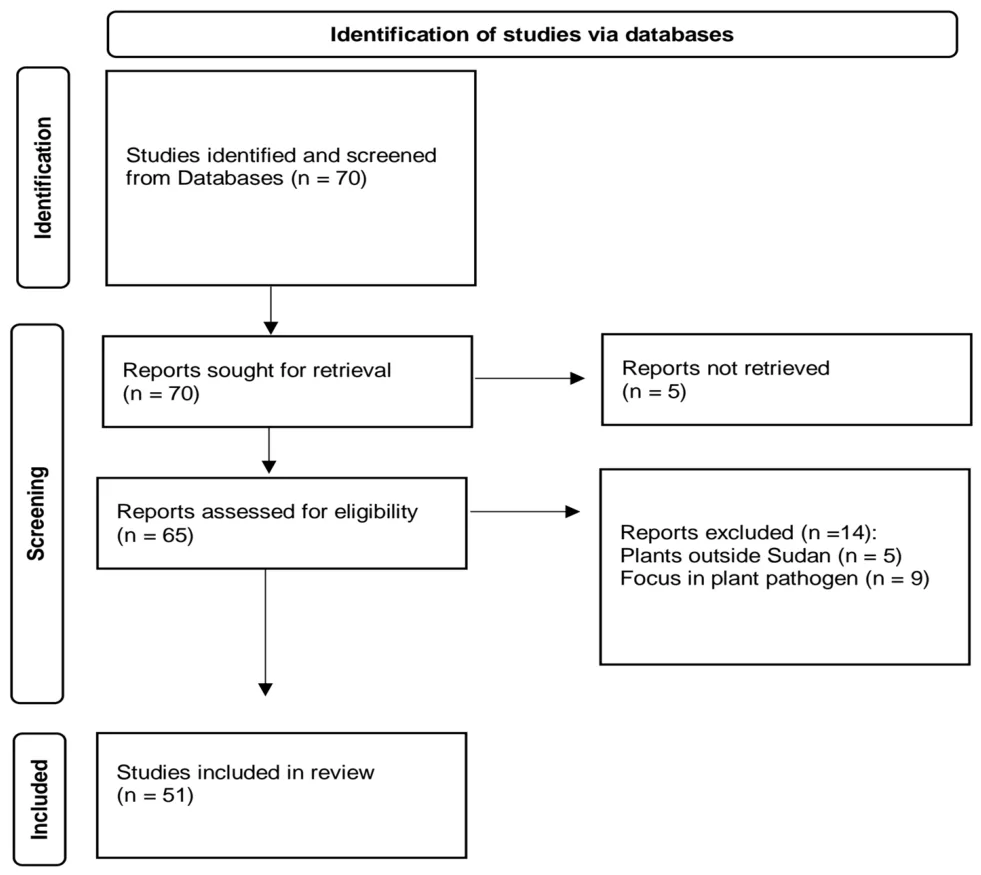
Flow chart demonstrating the search strategy and study selection for studies on Sudanese medicinal plants and their endophytes for antifungal activity.

A literature search was conducted up to 29 December 2025, covering publications between 2000 and 2025. However, select relevant studies were cited in the discussion strictly to provide a broader comparative context. To minimize selection bias and ensure data accuracy, title and abstract screening and subsequent data extraction were performed independently by two reviewers in duplicate. The literature selection criteria and multi-stage screening process are illustrated in Figure 1. Out of the 70 identified studies, 65 were assessed and 51 met all inclusion criteria. These 51 studies evaluated a total of 88 plant species across 51 families, as several individual studies screened multiple species and families (for example, one study screened up to 17 plant species).

Studies were included if they presented original research published as full peer reviewed articles, full conference proceedings, or conference abstracts (addressing novel or emerging subjects) evaluating the antifungal properties of Sudanese medicinal plants or their associated fungal endophytes against human fungal pathogens, published in English and indexed in major international electronic databases. Conversely, studies were excluded if they: (i) evaluated plant species not grown in Sudan; (ii) targeted exclusively non-human or agricultural pathogens, as the scope of this review is restricted to medical mycology; (iii) were published in local journals inaccessible through international databases due to repository server limitations; or (iv) lacked accessible full text.

Data extraction: Relevant data were organized using Microsoft Word, documenting parameters such as; botanical name, family, plant part utilized, extraction solvent, bioassay methodology, antifungal potency, target fungal species, and references (Table 1).

## 3. Anti-Fungal Activity of Sudanese Medicinal Plants

### 3.1. Antifungal Assays and Solvent Extraction

**Figure 2:**
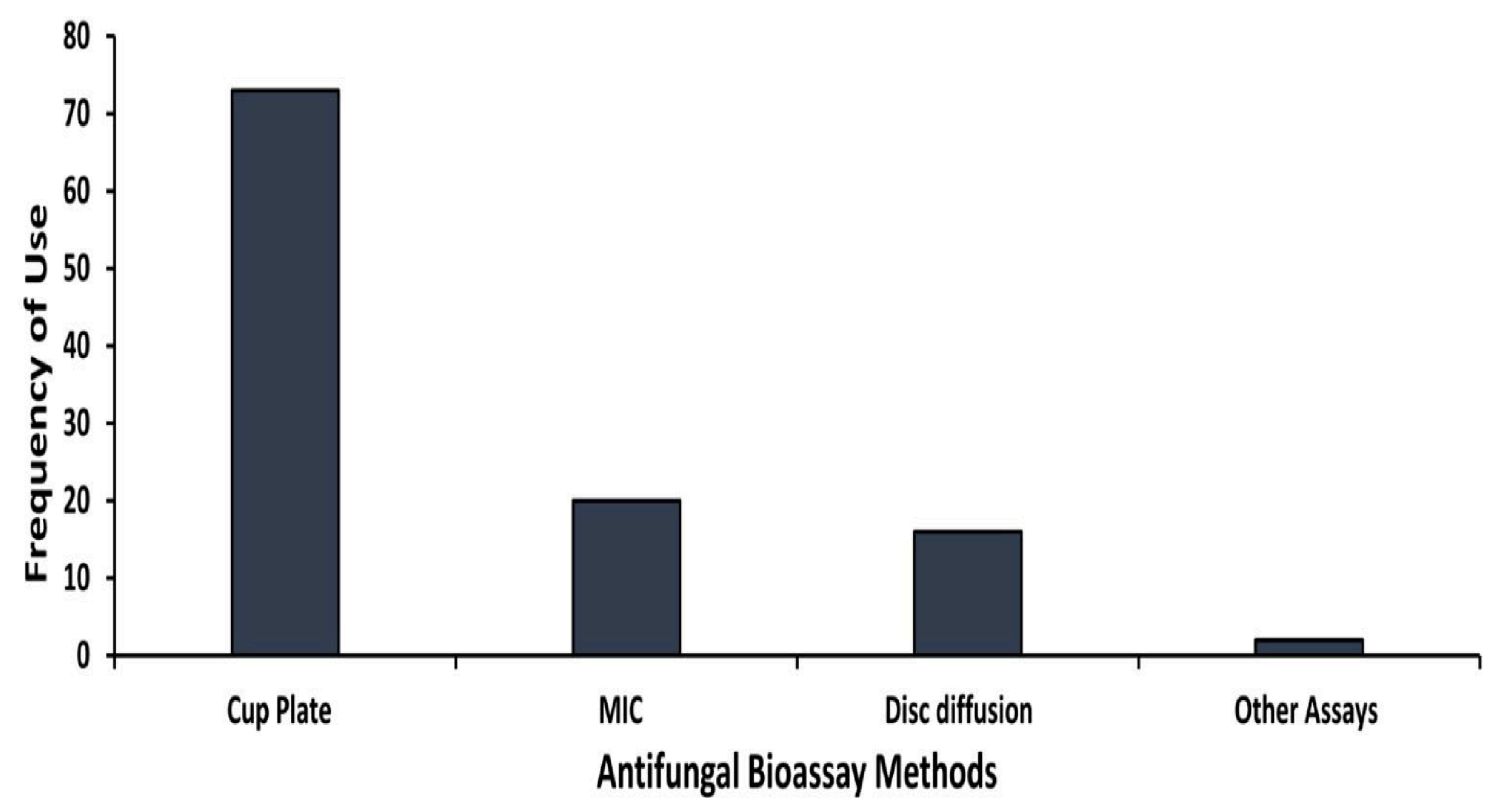
Distribution of experimental bioassay methods used to evaluate antifungal activity of Sudanese medicinal plants.

Antifungal studies on Sudanese medicinal plant extracts have been conducted using various methodologies. Figure 2 represents the proportion of studies and methods used to assess antifungal activity, mostly for the period 2000–2025. The most common methods used to investigate the antifungal activity of Sudanese medicinal plants are cup of agar diffusion, minimum inhibitory concentration (MIC) and disc diffusion. There are a few reports that mentioned mycelial fresh weight. Sasidharan et al. [32] reported that the main methods for in vitro antifungal screening are a diffusion method, MIC, minimum fungicidal concentration (MFC) and IC_50_ value on hyphal growth inhibition, as well as many other in vivo and in situ methods. The cup and disc diffusion studies were the first method of choice, possibly due to their simplicity and capacity to analyze a large number of test samples [33]. Even though disc or agar diffusion methodology is quick and a simple means of screening of antimicrobial activity, problems may arise when investigating oily samples [34,35]. Depending on the chemical nature of the particular sample, diffusion rates may produce inconsistent results [36]. Several papers reported results using both methods. The other antifungal method used in plant extract studies is the bio-autographic assay which was solely used to evaluate antifungal activity of isolated bioactive compounds from *Terminalia brownii* [37].

A critical evaluation of the experimental methodologies compiled in Supplementary Table S1 reveals substantial reporting gaps across the reviewed literature. A significant proportion of the included studies omitted essential assay parameters, such as solvent controls, explicit incubation temperatures/durations, and the number of biological replicates. These parameters are crucial for ensuring experimental reproducibility and allowing readers to accurately interpret antifungal activity. Among the studies that included positive controls, Nystatin and Clotrimazole were the most frequently used reference drugs, with fewer studies incorporating Fluconazole or Amphotericin B. Moreover, positive controls were applied at widely varying concentrations and units (e.g., 5 µg/mL vs. 50 µg/mL, or mg/disc vs. µg/mL).

Additionally, incubation conditions varied widely even across the same fungal species. These inconsistent incubation settings directly impact inhibition zone values, making direct comparisons between different studies difficult. Where reported, most studies utilized two or three replicates, but several omitted this detail altogether. It is important to emphasize that performing and reporting a minimum of three independent replicates (*n* = 3) is vital for statistical validity. Overall, the observed inconsistency in reporting highlights an urgent need for standardized protocols and adherence to international guidelines when evaluating the antifungal efficacy of plant extracts. Extract concentrations reported in Table 1 varied considerably across studies, ranging from 2.5 to 100 mg/mL. However, the majority of studies employing the agar well (cup) diffusion method used a fixed concentration of 50 mg/mL. Notably, several studies failed to report the volume or concentration of extract applied to the cup or paper disc. This lack of standardization represents a major methodological limitation in the existing literature on Sudanese medicinal plant antifungal research. Furthermore, the majority of the reviewed literature focuses on the preliminary screening of crude extracts, a finding that aligns with the observations of Abdalla and Abdalla [38]. These authors noted that research often remains at the screening stage due to a lack of specialized facilities and a deficiency in research funding. This trend shows the important need for increased investment and infrastructure support for scientific research in Sudan. Even though the country has a vast botanical wealth and its potential as a source for novel medicinal compounds, these systemic challenges hinder the progression from crude extract analysis to the isolation and characterization of pure bioactive compounds [39]. Among the studies reviewed, methanol (28.3%) was identified as the most prevalent solvent for extraction, followed by chloroform (26.2%) and water (23.6%). Methanol is widely preferred in antifungal research due to its high extraction yield and strong organic polarity. This property enables it to dissolve a broad spectrum of bioactive phytochemicals, ranging from highly polar compounds to medium- and low-polarity constituents. Furthermore, methanol’s low boiling point allows for rapid evaporation via rotary evaporators. However, it is not always the best choice, since solvent selection depends on the chemical nature of the target compounds. Water remains important for safety and traditional relevance, while ethanol offers a useful compromise between efficiency and lower toxicity. Chloroform and ethyl acetate are useful for less polar compounds, but their toxicity limits direct application. Essential oils (EO) are also important antimicrobial extracts, although their volatility and compositional variability can complicate standardization [40,41].

### 3.2. Plant Species, Botanical Families, and Utilized Organs

Table 1 summarizes the collected reports, detailing 78 plant species belonging to 43 families investigated for antifungal activity. This represents 25.3% of the 170 plant families documented in Sudan [1]. The top five families based on the number of species tested for antifungal activity were Fabaceae (n = 12), Malvaceae (n = 6), Asteraceae (n = 5), Euphorbiaceae (n = 5), and Combretaceae (n = 5). This pattern partially aligns with the global dataset summarized by Shivangani and Hariprasad [42], who reported that the most investigated plant families for antifungal activity are Lamiaceae, Fabaceae, Asteraceae, Myrtaceae, Apiaceae, Lauraceae, Rutaceae, Solanaceae, Euphorbiaceae, and Meliaceae. Notably, although Combretaceae is not among the ten most frequently studied antifungal families in that global review, its prominence in African traditional medicine, particularly for dermatological and antifungal applications, explains its strong representation in our findings. The majority of the plant families have been found to have antifungal properties that belong to Angiosperms (flowering plants). The only exception is the Salviniaceae family, which represents the Pteridophytes (seedless vascular plants). This taxonomic distribution reflects both the historical importance and pharmacological value of flowering plants in Sudanese traditional medicine.

**Figure 3:**
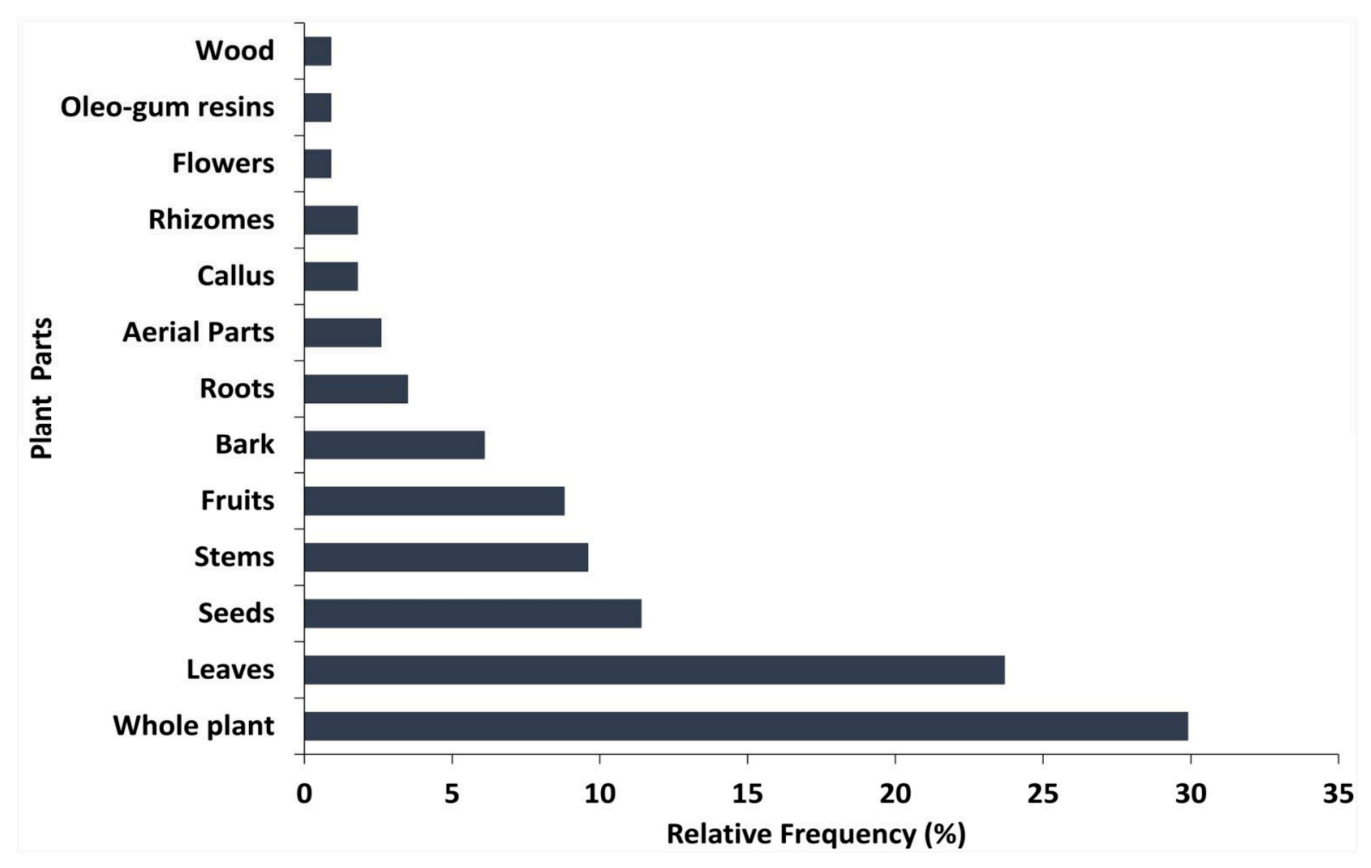
Plant parts reported to contain antifungal activity of Sudanese medicinal plants.

Figure 3 illustrates the distribution of various plant parts reported to possess antifungal activity. The analysis reveals that the whole plant represents the most frequently studied category, making up 29.9% of the literature. This is followed by leaves (23.7%) and seeds (11.4%). Other investigated components include stems (9.6%), fruits (8.8%), bark (6.1%), and roots (3.5%). While the whole plant ranks highest in this specific review, in general, antifungal research tends to focus more on leaves. This preference may be attributed to their environmental role; as leaves often produce a diverse array of antifungal secondary metabolites as a defense mechanism [42].

### 3.3. Targeted Fungal Pathogens

The reviewed literature identified various fungal targets, the majority of which belong to the phylum Ascomycota (sac fungi). The most frequently investigated species were *Candida albicans*, *Saccharomyces cerevisiae* and *Aspergillus niger*. This finding agrees with the report by Abdalla and Abdalla [38]. They suggested that these species received more attention due to their ease of culturing and general availability. Furthermore, they were likely randomly selected for initial screening studies. Specifically, *Aspergillus* was chosen as a typical fungus with significant medical and commercial importance, while *Candida* was selected as a typical yeast responsible for many superficial human infections. Additionally, a few studies evaluated the activity against *Aspergillus flavus* and several dermatophytes, including; *Epidermophyton floccosum*, *Microsporum audouinii*, and various *Trichophyton* species such as *T. rubrum*, *T. concentricum*, and *T. tonsurans*. Although plant-derived antifungal extracts typically show stronger activity against *Candida albicans* than against *A. niger* or *S. cerevisiae*, due to the thicker cell walls and complex conidial structures of filamentous fungi [43,44], our data in Table 1 shows an inverse pattern, with *A. niger* and *S. cerevisiae* being more susceptible. This indicates that fungal susceptibility is strongly influenced by extract composition, solubility, assay conditions, and plant source. Strain-specific resistance in *Candida*, including efflux activity, cell wall remodeling, biofilm formation, and growth-stage differences, can further reduce its sensitivity [45]. These findings emphasize the need to screen multiple fungal species and strains under varied conditions to identify robust antifungal leads.

### 3.4. Bioactive Components of Medicinal Plants

Some reports have mentioned general phytochemical screening and the majority indicate the presence of flavonoids, polyphenols, tannins and triterpenes which accumulate in the polar extracts such as *Portulaca oleracea* and *Combretum hartmannianum* [46,47]. *Lawsonia inermis*, a commercially important cultivated medicinal plant, demonstrated strong antifungal activity. The phytochemical analysis revealed the presence of anthraquinones and tannic acid in high concentrations [47,48]. Also, three reports confirm the activity of *Hydnora abyssinica* rhizome extract as a good antifungal which showed the presence of condensed tannins, cardiac glycosides, phenols, flavonoids and reducing sugars [49,50,51]. *Adansonia digitata* pulp extract showed antifungal activity, the HPLC analysis demonstrated that polyphenol constituents contained 18 compounds, four of them were major compounds: ellagic acid (8.36%), coumaric acid (8.84%), rosmarinic acid (7.37%) and cinnamic acid (8.59%) [52]. Variable antifungal activity has been reported for *Hyphaene thebaica*. While Elegami et al. [47] observed significant antifungal activity (IZ = 16–19 mm), Abdalla [53] reported that methanolic extracts were inactive. This discrepancy likely reflects differences in geographic origin, climatic conditions, harvesting environment, and extraction protocols, all of which influence the qualitative and quantitative profile of secondary metabolites. Environmental factors such as temperature, precipitation, drought stress, soil fertility, and light intensity are well known to modulate the biosynthesis and accumulation of bioactive compounds [54]. Furthermore, variations in extraction methods, assay protocols, and the specific fungal strains tested can yield divergent antifungal outcomes. Consequently, the size of inhibition zones in diffusion assays is influenced by numerous technical factors; thus, values reported across independent studies should be interpreted cautiously and compared only within the same methodological framework [55,56].

Five of the plant species belong to the family Combretaceae (*Combretum pentagonum*, *Combretum hartmannianum*, *Terminalia arjuna*, *Terminalia leiocarpa* and *T. brownii*) exhibiting potent antifungal activity. The bark methanolic extract of *T. arjuna* gave the lowest MIC value (4.25 mg/mL) against *Candida albicans*, whereas the stem methanolic extracts of *T. leiocarpa* gave the lowest MIC value (0.18 mg/mL) against *S. cerevisiae* [50]. Previous phytochemical investigation on various species of the Combretaceae, revealed that they are particularly rich in tannins, flavonoids and saponins. These bioactive chemical compounds were previously correlated with this antifungal activity [57,58,59,60]. Latte and Kolodziej [61], Mickymaray [62] and Savarirajan et al. [63] showed that tannins, flavonoids and saponins respectively were responsible for this antifungal activity. Among these bioactive chemical compounds Fyhrquist et al. [60] highlighted that tannins could be attractive due to their low toxicity to human beings. Similarly, a study conducted by Salih et al. [37] showed that leaf extracts of *T. brownii* demonstrated significant anti-*Candida* activity, with MIC values ranging from 312 to 2500 µg/mL. Sixty compounds were identified from an ethyl acetate fraction, including several gallotannins (di-, tri-, and tetra-), chebulinic acid, and ellagitannins such as corilagin and terflavin B, flavonoids such as apigenin and luteolin-7-O-glucoside. Among these isolated constituents, apigenin and β-sitosterol demonstrated the most potent growth inhibition. Notably, to the best of our knowledge, *T. brownii* represents the only Sudanese medicinal plant species that has undergone detailed bioassay-guided fractionation and compound isolation for antifungal activity during the specific period of review.

Sudan has a wide range of aromatic plants. Species characterized by their EO content, such as *Boswellia papyrifera* and *Pulicaria* spp., have demonstrated notable antifungal properties (MIC: 5 µg/mL, 6.25 mg/mL respectively). Compositional analysis via GC-MS revealed that the primary constituents of *P. crispa* EO are 1,4-di-tert-butylbenzene (22.81%) and caryophyllene (13.19%). In contrast, *P. undulata* oil is dominated by camphor (44.48%) and thymyl acetate (10.31%) [64]. Conversely, fixed oils extracted from seeds derived from other Sudanese medicinal plant species show a broad spectrum of efficacy; while seeds from *Acacia* spp., *Azadirachta indica*, and *Cannabis sativa* were found to be inactive [65,66,67], *Trigonella foenum-graecum* and *A. digitata* exhibited antifungal activity [68,69].

Evaluating the antifungal potential of Sudanese medicinal plants requires a systematic approach, progressing from broad comparative screenings across multiple species and families to detailed bioassays on selected target species. Bioactivity can vary markedly depending on whether evaluations focus on single plant parts (e.g., leaves, roots or bark) or combinations of organs, because phytochemical profiles and metabolite concentrations differ among tissues. Although phytochemical isolation aims to identify individual active molecules, bioactivity often arises from synergistic interactions among multiple secondary metabolites. As noted by Van Vuuren [33], a single isolated compound may lack the efficacy of the crude extract, in which diverse constituents act synergistically to produce an enhanced biological response. To the best of our knowledge, no studies have yet been conducted to elucidate the precise antifungal mechanisms of action of these Sudanese medicinal plants. A major limitation identified across the reviewed literature globally, is that approximately 80% of studies are restricted to phenotypic MIC determination without investigating underlying mechanisms of action. This gap severely limits the translational potential of promising extracts, because mechanistic elucidation is essential to distinguish target-specific antifungal effects from non-specific cytotoxicity or general membrane perturbation. Shivangani et al. [42] indicate that the most frequently reported mechanisms for crude extracts are ergosterol biosynthesis inhibition (37.5%), cell wall damage (15%), and cell membrane disruption (12.3%), whereas purified compounds most often target chitin synthase and mitochondrial dysfunction. This overall lack of mechanistic clarity constitutes a substantial impediment to the progress of plant-derived antifungal pharmaceuticals.

**Table 1:** In vitro antifungal assays of various Sudanese medicinal plant extracts.

Scientific Name	Family	Plant Part Analyzed	Solvent Used for Extraction	Assay Method (Concentration)	Target Fungi Species	Activity (ZI mm/MIC—MFC mg/mL)	References
*Hygrophila auriculata* (Schumach.) Heine (*Asteracantha longifolia* (L.) Nees)	Acanthaceae	Whole plant	Chloroform Methanol Water	Cup diffusion (50 mg/mL)	*Saccharomyces cerevisiae*, *Candida albicans*	-	[47]
*Aizoon canariense* L.	Aizoaceae	Whole plant	Chloroform Methanol Water	Cup diffusion (50 mg/mL)	*S. cerevisiae*, *C. albicans*	- 13, 12 -	[50]
*Limnophyton obtusifolium* (L.) Miq.	Alismataceae	Whole plant	Chloroform Methanol Water	Cup diffusion (50 mg/mL)	*S. cerevisiae*, *C. albicans*	13, - 15, 11 -	[47]
*Caroxylon imbricatum* (Forssk.) Moq. (*Salsola imbricata* Forssk.)	Amaranthaceae	Whole plant	Chloroform Methanol Water	Cup diffusion (50 mg/mL)	*S. cerevisiae*, *C. albicans*	11, - 12, - -	[50]
*Sclerocarya birrea* (A.Rich.) Hochst.	Anacardiaceae	Bark	Chloroform Ethanol Aqueous	Cup diffusion/MIC	*C.* *albicans*	- 14/0.37–1.5 -	[70]
*Ammi visnaga* (L.) Lam.	Apiaceae	Seeds	Chloroform Ethanol Aqueous	Cup diffusion/MIC	*C.* *albicans*	- 14/0.37–1.5 -	[70]
*Hyphaene thebaica* (L.) Mart.	Arecaceae	Fruit pulp	Methanol Ethanol Chloroform Methanol Water	Cup diffusion (500 mg/mL) Disc diffusion (100 mg/mL) Cup diffusion (50 mg/mL)	*Aspergillus niger*, *C. albicans* *A. niger*, *C. albicans* *S. cerevisiae*, *C. albicans*	- 9, 9 18, 14 19, 16 12, 13	[53] [71] [47]
*Hydnora abyssinica* A. Br.	Aristolochiaceae	Roots or Rhizomes	Chloroform Methanol Water	Disc diffusion (100 mg/mL)	*Epidermophyton* *floccosum*, *Microsporum audouinii*, *Trichophyton rubrum*, *Trichophyton concentricum*, *Trichophyton tonsurans*, and *C. albicans*.	Range 16.30–14.11 Range 16.15–14.00 Range 18.28–17.14	[49]
			Chloroform Methanol Water Ethanol	Cup diffusion/MIC (50 mg/mL) Cup diffusion (100 mg/mL)	*S. cerevisiae*, *C. albicans* *A. niger*, *C. albicans*	14, 12 26, 15/1.87, 7.50 15, 12 25.0, 23.0	[50] [51]
*Echinops longifolius* A. Rich.	Asteraceae	Whole plant	Chloroform Methanol Water	Cup diffusion (50 mg/mL)	*S. cerevisiae*, *C. albicans*	14, 11 16, 13 -	[50]
*Pulicaria crispa* (Forssk.) Oliv. *Pulicaria undulata* (L.) C.A.Mey.		Aerial parts (during flowering stage)	Methanol Hydrodistillation Methanol Hydrodistillation	Disc diffusion/MIC	*A. niger*, *C. albicans*	19, 20/6.25,50 21, 19/6.25,6.25 20, 21/6.25,6.25 22,23/6.25, 6.25	[64]
*Geigeria alata* Benth. & Hook.f. ex Oliv.		Leaves Roots Whole plant	Aqueous-methanol Chloroform Methanol Water	MIC Cup diffusion (50 mg/mL)	*C. albicans* *S. cerevisiae*, *C. albicans*	- 15, - 17, 14 -	[72] [47]
*Ceruana pratensis* Forssk.		Whole plant	Chloroform Methanol Water	Cup diffusion (50 mg/mL)	*S. cerevisiae*, *C. albicans*	16, - 20, 15 -, 14	[47]
*Cotula anthemoides* Lour.		Whole plant	Chloroform Methanol Water	Cup diffusion (50 mg/mL)	*S. cerevisiae*, *C. albicans*	19, 16 20, - -	[47]
*Echium rauwolfii* Delile	Boraginaceae	Whole plant	80% Ethanol	Cup diffusion (100 mg/mL)	*C. albicans*	25	[73]
*Brassica nigra* (L.) W.D. J. Koch	Brassicaceae	Seeds	Chloroform Ethanol Aqueous	Cup diffusion	*C. albicans*	-	[70]
*Rorippa indica* (L.) Hiern		Whole plant	Chloroform Methanol Water	Cup diffusion (50 mg/mL)	*S. cerevisiae*, *C. albicans*	- 12, - -	[47]
*Boswellia papyrifera* (Caill. ex Delile) Hochst.	Burseraceae	Oleo-gum resins	Petroleum ether Chloroform Methanol Hot water Hydrodistillation	Cup diffusion/MIC	*A. niger*, *A. flavus*, and *C. albicans*	13, -, 13/5, 5, 5 * 13, -, 14/5, 5, 5 -, -, 12/5, 5, 5 11, -, -/5, 5, 5 50,50,50/5, 5, 5	[74]
*Cannabis sativa* L.	Cannabaceae	Seeds Whole plant	Methanol Petroleum ether Methanol	Cup diffusion	*A. niger*, *C. albicans*	- - -, 13	[47]
*Cadaba farinosa* Forssk.	Capparaceae	Leaves	Chloroform 80% Methanol	Cup diffusion/MIC (10 mg/mL)	*A. niger*, *C. albicans*	- 12, -/range 2.5–5	[75]
*Maerua oblongifolia* (Forssk.) A. Rich.		Leaves Leaves Stem	Chloroform 80% Methanol Chloroform 80% Methanol Chloroform Methanol 80%	Cup diffusion/MIC (10 mg/mL) Cup diffusion/MIC	*A. niger*, *C. albicans* *A. niger*, *C. albicans*	15, -/range 2.5–5 15, -/range 2.5–5 14, -/range 2.5–5 15, -/range 2.5–5 12, 16/range 2.5–5 19, 13/range 2.5–5	[75] [76]
*Ceratophyllum demersum* L.	Ceratophyllaceae	Whole plant	Chloroform Methanol Water	Cup diffusion (50 mg/mL)	*S. cerevisiae*, *C. albicans*	-	[50]
*Combretum hartmannianum* Schweinf.	Combretaceae	Leaves Bark Stem Fruits Leaves	Chloroform Methanol Water Chloroform Methanol Water Chloroform Methanol Water Chloroform Methanol Water Ethanol	Cup diffusion/MIC (50 mg/mL) Cup diffusion (100 mg/mL)	*S. cerevisiae*, *C. albicans* *A. niger*, *C. albicans*	18, 15 28, 22/0.73, 5.90 18, 16/40, 40 15, 15 24, 20/0.42, 13.50 18, 13/26.97, 26.97 15,14 19,13 12, - 17, 16 30, 21/0.03, 3.98 22, 15/20.33, 20.33 23.5, 17.5	[47] [51]
*Terminalia leiocarpa* (DC.) Baill. (*Anogeissus schimperi* Hochst. ex Hutch. & Dalziel)		Fruits	Chloroform Methanol Water	Cup diffusion/MIC (50 mg/mL)	*S. cerevisiae*, *C. albicans*	17, 15 21, 20/0.18, 7.93 14, 13/0.55, 8.81	[50]
*Terminalia arjuna* (Roxb.) Wight. & Arn.		Bark	Chloroform Methanol Water	Cup diffusion/MIC (50 mg/mL)	*S. cerevisiae*, *C. albicans*	13, - 18, 19/0.23,4.25 13, 15/0.53, 8.59	[50]
*Terminalia brownii* Fresen.		Leaves Stem wood, Stem bark Roots Root bark	Hexane Dichloromethane Acetone Ethyl acetate Methanol Aqueous	Disc diffusion/MIC (50 mg/mL)	*C. albican*, *C. glabrata*, *C. tropicalis*, and *C. parapsilosis*	Range—to 38/312–2500^*^	[37]
*Combretum pentagonum* M. A. Lawson		Leaves Stem	Chloroform Methanol Water Chloroform Methanol Water	Cup diffusion/MIC (50- 5 mg/mL)	*S. cerevisiae*, *C. albicans*	- 25, 19/0.34, 5.57 15, 14/0.20, 6.52 - 23, 15/0.51, 8.25 15, 13/0.21, 13.72	[50]
*Cordia africana* Lam.	Cordiaceae	Leaves Stem Bark Fruits	Petroleum ether Chloroform Ethylacetate Methanol Water	Cup diffusion/MIC (100 mg/mL)	*A. niger*, *C. albicans*	Range—to 18 Range—to 26 Range—to 16 Range—to 18/ (MIC range 6.25–25)	[77]
*Citrullus amarus* Schrad. (*Citrullus lanatus* var. *citroides*)	Cucurbitaceae	Leaves Stem Fruits Seeds	n-Hexane Chloroform Ethanol	Cup diffusion (100 mg/mL)	*A. niger*, *C. albicans*	Range—to 37 Range—to 41 Range—to 41 Range—to 37	[78]
*Citrullus colocynthis* (L.) Schrad.		Aerial parts	80% Ethanol	Cup diffusion (100 mg/mL)	*C. albicans*	22	[73]
*Cucurbita maxima* Duchesne		Leaves Seeds	Ethanol	Cup diffusion (100–12.5 mg/mL)	*A. niger*, *C. albicans*	Range 18–14 Range 17–13	[79]
*Cyperus rotundus* L.	Cyperaceae	Whole plant	Ethanol	Cup diffusion (100 mg/mL)	*A. niger*, *C. albicans*	20, 26	[80]
*Euclea racemosa* subsp. *Schimperi* (A.DC.) F. White (*Euclea schimperi* (A. DC.) Dany Ogunim)	Ebenaceae	Leaves Stem	Chloroform Methanol Water Chloroform Methanol Water	Cup diffusion (50 mg/mL)	*S. cerevisiae*, *C. albicans*	12, - 20, 18 12, 13 - 16,14 -, 12	[47]
*Bergia suffruticosa* (Delile) Fenzl	Elatinaceae	Whole plant	Chloroform Methanol Water	Cup diffusion/MIC (50 mg/mL)	*S. cerevisiae*, *C. albicans*	15, - 25, 16 -	[50]
*Ricinus communis* L.	Euphorbiaceae	Leaves Seeds	Methanol	Cup diffusion (100 mg/mL)	*A. niger*, *C. albicans*	15, 14 -	[81]
*Euphorbia cuneata* Vahl.		Whole plant	Chloroform Methanol Water	Cup diffusion/MIC (50 mg/mL)	*S. cerevisiae*, *C. albicans*	13, 11 20, 14/0.60, - -	[50]
*Euphorbia scordiifolia* Jacq.		Leaves Stem	Chloroform Methanol Water Chloroform Methanol Water	Cup diffusion (50 mg/mL)	*S. cerevisiae*, *C. albicans*	18, 15 26, 17 19, 14 14, - 18, 13 -	[50]
*Euphorbia polyacantha* Boiss. (*Euphorbia thi* Schweinf.)		Whole plant	Chloroform Methanol Water	Cup diffusion (50 mg/mL)	*S. cerevisiae*, *C. albicans*	- 16, 14 -	[50]
*Jatropha aceroides* Hutch.		Stem	Chloroform Methanol Water	Cup diffusion (50 mg/mL)	*S. cerevisiae*, *C. albicans*	- 15, - -	[50]
*Acacia hamiltoniana* Maiden (*Acacia sieberiana* Tausch.) *Vachellia seyal* var. *fistula* (Schweinf.) *Kyal.* & *Boatwr*. (*Acacia seyal* var. *fistula* (Schweinf.) Baker) *Senegalia polyacantha* (Willd.) Seigler & Ebinger (*Acacia polyacantha* Willd.) *Vachellia nilotica* (L.) *P.J.H.Hurter* & *Mabb*. (*Acacia nilotica* (L.) Willd. ex Delile)	Fabaceae	Seeds Leaves	n-Hexane Ethanol	Cup diffusion (100–12.5 mg/mL) Cup diffusion (100–12.5 mg/mL)	*C. albicans* *A. niger*, *C. albicans*	- - - 22–18, 21–16	[65] [82]
*Sesbania leptocarpa* Dc.		Leaves	Ethanol	Cup diffusion (100 mg/mL)	*A. niger*, *C. albicans*	-	[51]
*Trigonella foenum-graecum* L.		Seeds Callus	Petroleum ether Methanol Petroleum ether Methanol	Disc diffusion (250–125/mL)	*A. niger*, *C. albicans*	20–15, 17–15 -, 8–7 -	[68]
*Tephrosia purpurea* (L.) Pers.		Whole plant	Chloroform Methanol Water	Cup diffusion (50 mg/mL)	*S. cerevisiae*, *C. albicans*	11, - 18, 13 -	[50]
*Senna occidentalis* (L.) Link		Leaves	Chloroform 80% Methanol	Cup diffusion/MIC (10 mg/mL)	*A. niger*, *C. albicans*	-, 15/range 2.5–5 17, 15/range 2.5–5	[75]
*Aeschynomene indica* L.		Leaves + Fruits	Chloroform Methanol Water	Cup diffusion (50 mg/mL)	*S. cerevisiae*, *C. albicans*	- 13, - -	[47]
*Dichrostachys cinerea* (L.) *Wight* & *Arn*.		Stem Leaves	Water Methanol Water Methanol	Cup diffusion	*C.* *albicans*	19.94 19.54 20.17 22.14	[83]
*Indigofera spinosa* Forssk.		Whole plant	Chloroform Methanol Water	Cup diffusion (50 mg/mL)	*S. cerevisiae*, *C. albicans*	15, 11 16, 13 -	[47]
*Neptunia oleracea* Lour.		Whole plant	Chloroform Methanol Water	Cup diffusion (50 mg/mL)	*S. cerevisiae*, *C. albicans*	12, - 15, 11 -	[50]
*Najas pectinata* (Parl.) Magnus	Hydrocharitaceae	Whole plant	Chloroform Methanol Water	Cup diffusion (50 mg/mL)	*S. cerevisiae*, *C. albicans*	17, - 18, 12 -	[50]
*Leucas urticifolia* (Vahl) Sm.	Lamiaceae	Whole plant	Chloroform Methanol Water	Cup diffusion (50 mg/mL)	*S. cerevisiae*, *C. albicans*	14, - 15, 11 -	[50]
*Coleus barbatus* var. *barbatus* (*Plectranthus barbatus* Andr.)		Whole plant	Chloroform Methanol Water	Cup diffusion (50 mg/mL)	*S. cerevisiae*, *C. albicans*	- 19, 13 -	[50]
*Plicosepalus acaciae* (Zucc.) Weins. et Polhill.	Loranthaceae	Leaves Stems	Chloroform Methanol Water	Cup diffusion (50 mg/mL)	*C. albicans*	- 17, 14 15, 11	[84]
*Lawsonia inermis* L.	Lythraceae	Leaves Leaves	Chloroform Methanol Water Chloroform Methanol Water	Disc diffusion (25–75 mg/mL) Cup diffusion/MIC (50 mg/mL)	*E. floccosum*, *M. audouinii*, *T. rubrum*, *T. concentricum*, *T. tonsurans*, and *C. albicans* *S. cerevisiae*, *C. albicans*	Range 10.51–15.95 24, 12 25, 20/0.23, 7.41 24, 15	[48] [47]
*Adansonia digitata* L.	Malvaceae	Fruit pulp Fruit powder Leaves Seeds	Ethanol 80% Ethanol Ethanol Hexane	Disc diffusion (100 mg/mL) Cup diffusion/MIC Cup diffusion (100–12.5 mg/mL) Disc diffusion (100 mg/mL)	*A. niger*, *C. albicans* *A. niger*, *C. albicans* *A. niger*, *C. albicans* *C. albicans*	9, 13 11, 21/0.13–0.46, 0.06–0.16 18–14, 17–13 18	[71] [52] [79] [69]
*Corchorus tridens* L.		Aerial parts	n-Hexane Chloroform Ethylacetate Methanol	Disc diffusion (10 mg/mL)	*A. niger*, *C. albicans*	16, 15 -, 12 18, 13 17, 19	[85]
*Hibiscus sabdariffa* L.		Seeds Leaves Seeds Flowers	n-Hexane Ethanol Methanol Ethanol	Disc diffusion (100–12.5 mg/mL) Cup diffusion/MIC (100 mg/mL) Disc diffusion (100 mg/mL)	*C. albicans* *A. niger*, *C. albicans* *A. niger*, *C. albicans*	Range 16.5–15 Range 12–9 17, 24/25, 6.25 - 10, 11	[86] [81] [71]
*Abutilon pannosum* (G.Forst.) Schltdl.		Whole plant	Chloroform Methanol Water	Cup diffusion (50 mg/mL)	*S. cerevisiae*, *C. albicans*	15, - 13, 12 -	[47]
*Hibiscus vitifolius* L.		Whole plant	Chloroform Methanol Water	Cup diffusion (50 mg/mL)	*S. cerevisiae*, *C. albicans*	- 14, 12 -, 13	[47]
*Waltheria indica* L.		Whole plant	Methanol Chloroform Water	Cup diffusion (50 mg/mL)	*S. cerevisiae*, *C. albicans*	- 16, 14 -	[47]
*Azadirachta indica* L.	Meliaceae	Fruits	n-Hexane	Cup diffusion	*C. albicans*	-	[66]
*Ekebergia capensis* Sparrm.		Whole plant	80% Ethanol	Cup diffusion (100 mg/mL)	*C. albicans*	28	[73]
*Glinus lotoides* L.	Molluginaceae	Whole plant	Chloroform Methanol Water	Cup diffusion (50 mg/mL)	*S. cerevisiae*, *C. albicans*	17, 15 - -	[50]
*Ficus vasta* Frossk	Moraceae	Leaves	Ethanol	Cup diffusion (100 mg/mL)	*A. niger*, *C. albicans*	21, 19	[51]
*Moringa oleifera* Lam.	Moringaceae	Seeds	Aqueous Methanol	Disc diffusion (5–40%)	*A. niger*, *C. albicans*	Range 11 to 15, 11 -	[87]
*Ximenia americana* L.	Olacaceae	Bark Leaves Roots Stems	Chloroform Methanol Water	Cup diffusion/MIC (100 mg/mL)	*A. niger*, *C. albicans*, *S. cervisiae*	Range—to 27 (MIC range—to 0.29)	[88]
*Ludwigia erecta* (L.) H.Hara (*Jussiaea erecta* L.)	Onagraceae	Whole plant	Chloroform Methanol Water	Cup diffusion/MIC (50 mg/mL)	*S. cerevisiae*, *C. albicans*	15, - 27, 20 17, 12	[50]
*Ludwigia adscendens* subsp. *Adscendens* (*Jussiaea repens* L.)		Whole plant	Chloroform Methanol Water	Cup diffusion (50 mg/mL)	*S. cerevisiae*, *C. albicans*	- 20, 15 -	[50]
*Polygala irregularis* Boiss.	Polygalaceae	Whole plant	Chloroform Methanol Water	Cup diffusion (50 mg/mL)	*S. cerevisiae*, *C. albicans*	13, - - -	[50]
*Portulaca oleracea* L.	Portulacaceae	Aerial parts	n-Hexane Chloroform Ethyl acetate Methanol	Disc diffusion (10 mg/mL)	*A. niger*, *C. albicans*	15, 11 17, 13 12, 18 17, 19	[46]
*Nigella sativa* L.	Ranunculaceae	Seeds	Ethanol	Cup diffusion (100–12.5 mg/mL)	*A. niger*, *C. albicans*	Range 25–19, 21–18	[89]
*Ziziphus spina Christi* (L.) Desf.	Rhamnaceae	Fruit pulp Leaves	Ethanol n-Hexane Ethyl acetate Methanol	Disc diffusion (100 mg/mL) Cup diffusion (100–12.5 mg/mL)	*A. niger*, *C. albicans* *A. niger*, *C. albicans*	10, 7 -	[71] [90]
*Azolla nilotica* Dcne.	Salviniaceae	Whole plant	Chloroform Methanol Water	Cup diffusion (50 mg/mL)	*S. cerevisiae*, *C. albicans*	12, - 15, 11 -	[50]
*Datura stramonium* L.	Solanaceae	Leaves Callus	Petroleum ether Methanol	Cup diffusion	*A. niger*, *C. albicans*	- 12, 14	[91]
*Solanum nigrum* L.		Leaves	Chloroform 80% Methanol	Cup diffusion/MIC (10 mg/mL)	*A. niger*, *C. albicans*	15, 15/range 2.5–5 19, 15/range 2.5–5	[75]
*Cyphostemma cyphopetalum* (Fresen.) Desc. ex. Wild & R.B.Drumm. (*Cissus cyphopetala* Fresen)	Vitaceae	Whole plant	Chloroform Methanol Water	Cup diffusion (50 mg/mL)	*S. cerevisiae*, *C. albicans*	16, 14 17, 14 -	[47]
*Cissus quadrangularis* L.		Whole plant	Chloroform Methanol Water	Cup diffusion (50 mg/mL)	*S. cerevisiae*, *C. albicans*	- 17, 15 -, 13	[47]
*Curcuma longa* L.	Zingiberaceae	Rhizomes	Ethanol	Cup diffusion (100–12.5 mg/mL)	*C. albicans*, *C.* spp.	Range 10–15, Range 13–17	[92]

-: No antifungal activity against the tested microorganism, MIC: Minimum Inhibitory Concentration, MFC: Minimum fungicidal concentration, ZI: Zone of inhibition, *: µg/mL. World flora online: https://www.wfoplantlist.org/ (2025) nomenclature system was followed. Scientific names (in parentheses) are as mentioned by the author in the original publication.

## 4. Anti-Mycetoma Activity of Sudanese Medicinal Plants

A dedicated focus on mycetoma is warranted because Sudan represents a global hyper-endemic epicenter for eumycetoma (*M. mycetomatis*), a WHO-designated Neglected Tropical Disease characterized by severe tissue destruction and therapeutic resistance [24]. Given these distinct clinical challenges and the specialized bioassay protocols required for anti-mycetoma screenings compared to general opportunistic fungi, plant species evaluated against *M. mycetomatis* are synthesized in a separate standalone subsection.

The fungus *M. mycetomatis* is the most common causative agent world-wide [23]. For actinomycetoma success rates up to 90% are achieved when treated with antibiotics [93,94]. Treating eumycetoma necessitates a dual approach of surgical removal combined with an extended course of antifungal therapy, typically using Itraconazole. Despite this rigorous treatment regimen, recurrence rates are high, ranging from 25 to 50%. These relapses often lead to worse health complications and require limb amputation in up to 25% of cases [95,96]. Treatment with antifungal agents has given a mixed response, with only Itraconazole being effective. These techniques are relatively expensive and cannot be afforded by people with a weak economic background [97]. The limited documentation of African plants employed in the treatment of mycetoma may be attributed to either the efficacy of modern antibiotics or insufficient research conducted in endemic regions. Consequently, additional research is imperative to elucidate the African folklore related to the management of this disease. In light of this situation, it is essential to implement herbal remedies to mitigate the related health and economic burdens [98]. This section of the review, along with Table 2, concentrates on various medicinal plants from Sudan that demonstrate considerable effectiveness against mycetoma through antifungal properties. Ezaldeen et al. [99] reported a cross-sectional study of 311 eumycetoma patients at the Mycetoma Research Center (MRC), University of Khartoum, Sudan, which indicated that 42.4% of the patients utilized herbal medicines during their illness. *Moringa oleifera*, *V. nilotica*, *Citrullus colocynthis*, and *Cuminum cyminum* were the major species that were used. However, most users reported no therapeutic benefit, and 29.3% experienced complications. The high reliance on herbal remedies likely reflects dissatisfaction with current antifungal treatments. This shows that there is a need for more public health regulation and more research on the pharmacological effects of common plants. Kunna et al. [100] also stated that a cross-sectional study of 389 confirmed mycetoma patients at the MRC showed that all of the participants had used traditional medicine at some point during their treatment. Two-thirds initially consulted traditional healers, predominantly religious healers (fakis) and herbalists. Herbal medicine was the most common treatment that the specialist healers gave. Due to many reports of patients using traditional remedies, several Sudanese researchers, often in partnership with international research institutions have undertaken studies to empirically validate the efficacy of indigenous medicinal plants against mycetoma. Abd Algaffar and Khalid [101] investigated the roots of *Tinospora bakis* and the rhizomes of *Curcuma longa*, which exhibited potential efficacy against *M. mycetomatis*. The chloroform and methanol-acetone mixture extracts of *T. bakis* had MICs of 78.1 and 39.1 µg/mL, respectively. The 70% ethanolic extract of *Curcuma longa* had a MIC of 39.1 µg/mL. The observed activity is attributed to bioactive compounds such as berberine in *T. bakis* and curcumin in *Curcuma longa*, indicating their potential as natural antifungal agents for mycetoma treatment. In addition, the chloroform root extract of *Haplophyllum tuberculatum* demonstrated antifungal activity against *M. mycetomatis* in a resazurin microplate assay. HPLC-guided fractionation resulted in the isolation of two active lignans, nectandrin B and tetrahydrofuroguaiacin B, recognized as the principal antifungal components [102]. Ahmed et al. [103] found that ethanolic extracts of both fermented and non-fermented *Senna obtusifolia* leaves, as well as their solvent fractions, demonstrated considerable antifungal activity against *M. mycetomatis* (MIC = 39.1 µg/mL). Comparative chemical analysis (TLC, RP-HPLC-DAD, and LC–MS/MS) demonstrated that fermentation caused significant qualitative and quantitative alterations in polyphenolic composition, particularly converting flavones into phenolic acids. These chemical modifications may contribute to the observed antifungal efficacy and highlight the influence of fermentation on the bioactivity of *S. obtusifolia*.

The methanolic extract of *T. brownii* leaves and its fractions exhibited significant antifungal activity against *M. mycetomatis*. The methanolic crude extract had a MIC of 78.1 µg/mL, while the chloroform and ethyl acetate fractions exhibited stronger inhibition (MIC < 39.1 µg/mL). LC–MS/MS analysis identified various bioactive phenolics and flavonoids, including ellagic acid, catechin-3-O-gallate, isoorientin, myricetin-3-O-hexose gallate, and prenylated stilbenes, which are likely responsible for the observed antifungal activity [104]. Elfadil et al. [105] assessed the antifungal efficacy of methanolic extracts from seven Sudanese medicinal plants against *M. mycetomatis*, with *B. papyrifera*, *Vachellia oerfota* var. *oerfota* and *N. sativa* exhibiting notable effects. *Boswellia papyrifera* showed the strongest (MIC_50_ = 1 µg/mL), particularly in its methanol and ethyl acetate fractions. GC–MS analysis identified β-amyrin, β-amyrone, β-sitosterol, and stigmatriene, with the latter displaying the highest activity (MIC_50_ = 32 µg/mL). This MIC is much higher than the MIC of the whole crude extract (1 μg/mL), indicating that other compounds or a combination of compounds has a better ability to inhibit *M. mycetomatis* growth. The enhanced activity of the crude extracts suggests possible synergistic interactions. On the other hand, EO extracted from *G. alata*, *Myristica fragrans*, *Pimpinella anisum*, *Syzygium aromaticum*, and *Thymus vulgaris* exhibited potent in vitro antimycetomal activity against *M. mycetomatis*, with MICs ranging from 0.004–0.125% *v*/*v*. Among these, *P. anisum* EO showed the most promising efficacy, significantly enhancing survival in infected *Galleria mellonella* larvae without toxicity. GC-MS analysis revealed that the major component of the active *P. anisum* EO is trans-anethole (76.87%) [106].

Therefore, a paucity of research exists concerning the impact of Sudanese medicinal plants on eumycetoma, with the majority of studies emerging after the year 2000. This part of the review identifies 16 plant species spanning 14 families that have been evaluated for their anti-eumycetoma properties. The broth microdilution method was the primary technique employed to determine in vitro activity against *M. mycetomatis*. Various plant parts, including roots, rhizomes, bark, gum resins, and seeds, were extracted using organic solvents of differing polarities, and EO were obtained by hydrodistillation. In our dataset (Table 2), hydrodistilled EO and methanolic extracts generally showed the strongest antimycetoma activity, indicating that volatile terpenoids and semi-polar metabolites are particularly relevant in this context. Notably, *B. papyrifera* extracts and *P. anisum* EO exhibited potent and promising efficacy against the eumycetoma pathogen.

The reported activity of several Sudanese medicinal plants is promising given the limited efficacy of existing eumycetoma therapies. Thus, MIC values below 50 μg/mL for key Sudanese extracts and fractions are encouraging compared to other natural product leads. However, since crude extracts are chemically complex and may exert synergistic effects, their MIC values cannot be directly equated to pure antifungals like Itraconazole or Fluconazole. Rather, they serve as preliminary indicators of bioactive leads requiring further isolation and characterization [55,107]. In addition, comparing findings across regions remains challenging due to the limited number of studies in Sudan and neighboring areas, most of which lack standardized fractionation protocols. Ultimately, detailed investigations into their mechanisms of action are required before these plant extracts can be developed into viable therapeutic candidates [105]. Medicinal plants may exert anti-mycetoma effects through both pathogen directed and host directed mechanisms. In *B. papyrifera*, antifungal activity against *M. mycetomatis* has been attributed to triterpenoid-rich extracts and may reflect, at least in part, disruption of fungal viability, although the precise molecular target remains unresolved. Importantly, the same extract may also be valuable because mycetoma is a chronic inflammatory disease; thus, anti-inflammatory and wound-healing properties could help reduce the persistent inflammatory tissue response surrounding the grain. More generally, plant-derived metabolites may compromise fungal membrane integrity, interfere with cell wall biosynthesis, impair metabolic processes, and inhibit biofilm formation or other protective structures that support persistence within host tissue [105]. A more defensible mechanistic interpretation is that anise oil may act through broad antifungal stress, membrane disruption, and possible host-modulating effects rather than a single confirmed molecular target [106]. Other medicinal plants employ multifaceted strategies against mycetoma pathogens. Bioactive secondary metabolites such as alkaloids, terpenoids, and phenolics can disrupt plasma membrane integrity, induce metabolic dysfunction, and trigger apoptosis. Interestingly, certain natural compounds, such as synthetic cinnamon oil derivatives (CIN-102), may not reduce grain size or burden directly; instead, they potentiate host immune responses by stimulating the rapid encapsulation of fungal grains by host immune cells, thereby localizing and neutralizing the infection [108].

**Table 2:** Antimycetomal activity of Sudanese medicinal plants extracts.

Scientific Name	Family	Plant Part Analyzed	Method for Extraction	Antimycetomal Activity Against *M. mycetomatis*	References
*Pimpinella anisum* L.	Apiaceae	Fruits	Hydrodistillation	MIC/MFC (%*v*/*v*) of three *M. mycetomatis* strains ranged from 0.008–0.125 and 0.031–0.125%.	[106]
*Geigeria alata Benth.* & *Hook.f*. ex Oliv.	Asteraceae	Roots Aerial parts	Hydrodistillation	Root EO. MIC/MFC (%*v*/*v*) of three *M. mycetomatis* strains ranged from (<0.008–0.016/0.016–0.16%). Areal part EO 0.016–0.031/0.016–0.125%.	[106]
*Boswellia papyrifera* (Caill. ex Delile) Hochst.	Burseraceae	Gum resin	Hexane Ethyl acetate Methanol	Showed potent activity with MIC ranging from 1–128 μg/mL.	[105]
*Terminalia brownii* Fresen.	Combretaceae	Leaves	Petroleumether Chloroform Ethyl acetate 80% Methanol	Methanolic crude extract showed activity with an MIC of 78.1 μg/mL, while the chloroform and ethyl acetate fractions exhibited significant activity with MIC < 39.1 μg/mL.	[104]
*Senna obtusifolia* (L.) H.S.Irwin & Barneby (*Cassia obtusifolia* L.)	Fabaceae	Leaves (fermented and non-fermented)	Petroleum ether Chloroform Ethyl acetate 80% Ethanol	Both the fermented and non-fermented and their fractions exhibited an MIC of 39.1 μg/mL.	[103]
*Vachellia oerfota* var. *oerfota* (*Acacia nubica* Benth.)		Root bark	Hexane Ethyl acetate Methanol	Methanol and hexane extracts were active with MIC ranging from 1 to 4 μg/mL.	[105]
*Thymus vulgaris* L.	Lamiaceae	Leaves	Hydrodistillation	MIC/MFC (%*v*/*v*) of three *M. mycetomatis* strains ranged from (0.004–0.016/0.008–0.016).	[106]
*Cinnamomum verum* J. Presl.	Lauraceae	Stem peel	Hexane Ethyl acetate Methanol	Only methanol crude extract inhibited fungal growth with MIC value 25 µg/mL.	[105]
*Tinospora bakis* (A. Rich.) Miers	Menispermaceae	Roots	Chloroform Methanol and Acetone mix	Chloroform has MIC: 78.1 µg/mL methanol and acetone mix has MIC 39.1 µg/mL.	[101]
*Myristica fragrans* Houtt.	Myristicaceae	Seeds	Hydrodistillation	MIC/MFC (%*v*/*v*) of three *M. mycetomatis* strains ranged from (0.008–0.031/0.016–0.063%).	[106]
*Syzygium aromaticum* (L.) Merr. & L.M.Perry (*Eugenia caryophyllus*)	Myrtaceae	Flower buds	Hydrodistillation	MIC/MFC (%*v*/*v*) of three *M. mycetomatis* strains ranged from (<0.008–0.063/<0.008–0.063%).	[106]
			Hexane Ethylacetate Methanol	Only methanol crude extract inhibited fungal growth with MIC value 50 µg/mL.	[105]
*Piper nigrum* L.	Piperaceae	Fruits	Hexane Ethyl acetate Methanol	Only methanol crude extract inhibited fungal growth with MIC value 25 µg/mL.	[105]
*Nigella sativa* L.	Ranunculaceae	Seeds	Hexane Ethyl acetate Methanol	Methanol and hexane extracts were active with MIC ranging from 1 to 4 μg/mL.	[105]
*Haplophyllum tuberculatum* (Forssk.) A.Juss.	Rutaceae	Roots	Chloroform	Active (The MIC value was not mentioned by the author).	[102]
*Curcuma longa* L.	Zingiberaceae	Rhizomes	Ethanol 70% Ethyl acetate	Ethanol with MIC of 39.1 ug/mL Ethyl acetate with MIC of 156 µg/mL.	[101]
*Zingiber officinale* Roscoe		Rhizomes	Hexane Ethyl acetate Methanol	Only methanol crude extract inhibited fungal growth with MIC value of 12.5 µg/mL.	[105]

MIC: Minimum inhibitory concentration, MFC: Minimum fungicidal concentration, EO: essential oil. Scientific names (in parentheses) are as mentioned by the author in the original publication.

## 5. Antifungal Activity of Endophytic Fungi

Endophytic fungi serve as a prolific reservoir of antifungal agents, synthesizing metabolites with potent activity against fungal pathogens. Their antagonistic effects are mediated through the production of primary and secondary metabolites, together with volatile organic compounds, which suppress pathogen growth via multiple biochemical mechanisms [109,110]. The ability of endophytes to synthesize these metabolites is thought to have evolved through their long-term mutualistic association with host plants. By limiting the establishment of invading pathogens and competing microorganisms, endophytes enhance host survival while maintaining their own ecological niche within plant tissues. Continuous co-evolution with medicinal plants has likely contributed to the remarkable structural diversity and biological activity of endophyte-derived metabolites, as these microorganisms adapt to the host’s chemical composition and environmental pressures. Consequently, medicinal plants constitute an important reservoir of endophytic fungi capable of producing structurally diverse antifungal compounds with considerable pharmaceutical potential [111,112]. From an applied perspective, endophytic fungi provide several advantages over direct extraction of bioactive compounds from medicinal plants. Unlike plant tissues, endophytes can be cultured under controlled laboratory conditions and propagated through microbial fermentation, allowing a reliable and scalable production of valuable metabolites. This strategy reduces dependence on seasonal availability, geographical variation, and intensive harvesting of medicinal plants, thereby contributing to the conservation of natural plant populations. In addition, recent advances in fermentation technology, metabolic engineering, and genome-guided natural product discovery have created new opportunities to improve metabolite yields and accelerate the development of endophyte-derived antifungal agents for pharmaceutical applications [113,114].

In Sudan, only a limited number of medicinal plants have been investigated for endophytic microorganisms. Within this subset of reviewed literature, three out of the seven published studies evaluated the antifungal activity of isolated endophytes. Across these primary studies, four Sudanese medicinal plant species were collected for endophyte isolation, yielding a total of 25 endophytic fungal isolates, predominantly belonging to the genera *Curvularia* (5 isolates) and *Aspergillus* (4 isolates). Plant leaves and twigs constituted the main source of endophytes. Ethyl acetate was the most frequently used extraction solvent, followed by methanol. Ethyl acetate is widely used for extracting endophytic fungal metabolites in bioassay screening [115]. Its intermediate polarity allows efficient recovery of a broad range of semi-polar bioactive compounds, and it is easy to evaporate, forms clean phase separations with aqueous broths, and is less toxic than many chlorinated solvents, making it particularly suitable for natural products–based antifungal discovery. Across the reviewed literature, the referenced fungal species consistently utilized for antifungal assays were *Candida albicans* and *A. niger*. These were frequently employed as standard models to evaluate the antifungal efficacy of Sudanese plant extracts and their endophytes (Table 3). The trends of antifungal activity for endophyte derived extracts show higher growth inhibition against *Candida albicans* than against *A. niger*. This pattern aligns with broader antifungal screening trends in which yeasts are often more susceptible than filamentous fungi, although it is not universal and depends on the endophyte strain, host plant, extraction parameters, and assay conditions. In general, the antifungal activity varied depending on the endophyte isolate, plant host, and the tested pathogens.

More recently, Kheir et al. [116] isolated 10 endophytic fungi from *V. nilotica*, whose methanolic extracts demonstrated broad-spectrum antimicrobial activity, particularly against fungi. Some isolates, such as *Curvularia* sp. (isolated from twigs), *Chaetomium* sp. (isolated from leaves), and *Alternaria* sp. (isolated from fruits), exhibited stronger antifungal activity against *A. niger* (ZI = 20.6, 20.0, and 18.3 mm, respectively) than the extracts from their corresponding host organs (ZI = 13.3, 11.3, and 11.3 mm). The first and last of these isolates also inhibited *Candida albicans* (ZI = 20.6 and 22.7 mm) with an effect comparable to the host plant extracts (ZI = 20.7 and 23.3 mm). They also profiled the methanolic extracts of endophytes isolated from *V. nilotica* and found gallic acid and naringenin to be the major constituents. They observed that extracts rich in gallic acid lacked naringenin and vice versa. Leaf-derived endophytes were enriched in gallic acid; fruit-derived ones were rich in naringenin, and twig-derived isolates varied between the two. To enable a more rigorous evaluation of the reported antifungal efficacy of endophytic fungi, available methodological parameters were compiled in Supplementary Table S2. Where specified, Nystatin served as the positive control. However, a critical limitation highlighted by this Supplementary Table S2 is the frequent omission of essential experimental details in primary studies, including unstated solvent controls, missing replication parameters, and unspecified incubation conditions. This lack of standardized reporting underscores the need for consistent methodological frameworks in future antifungal evaluations of endophytes associated with Sudanese medicinal plants. Overall, this section of the review indicates that Sudanese medicinal plants harbor promising endophytic fungi that could serve as valuable sources for new antifungal compounds with potential industrial and biotechnological applications.

Nevertheless, the small number of studies available reflects the early stage of endophyte research in Sudan rather than the true diversity of its medicinal flora. Research on Sudanese medicinal plants has traditionally emphasized the phytochemistry and biological activities of plant tissues, whereas investigations of their associated microbiota have been relatively scarce, largely because endophyte isolation, taxonomic identification, and metabolite characterization require specialized methodologies and multidisciplinary expertise. Importantly, although only four medicinal plant species have been examined to date, every species investigated yielded cultivable fungal endophytes with measurable antifungal activity, suggesting that these microorganisms remain an under explored biological resource. Expanding endophyte research to include a broader range of Sudanese medicinal plants is therefore justified and may facilitate the discovery of novel antifungal metabolites. Moreover, the use of endophytic fungi as microbial producers provides a sustainable alternative to extensive harvesting of medicinal plants, enabling renewable production of valuable natural products through fermentation while contributing to biodiversity conservation and reducing pressure on wild plant populations [117,118].

**Table 3:** Antifungal activity of endophytic extracts isolated from Sudanese medicinal plants.

Scientific Name of Plant Host	Plant Family	Host Plant Organ	Endophytes	Solvent for Extraction	Assay Method	Antifungal Activity Against (ZI mm)		References
						*C. albicans*	*A. niger*	
*Kigelia africana* (Lam.) Benth.	Bignoniaceae	Leaves Stem Bark	*Cladosprium* sp. *Aspergillus flavus* *Curvularia lunta* *Curvularia* sp. *Aspergillus* sp.	Ethyl acetate	Cup diffusion	14.0 12.0 16.0 16.0 17.0	14.0 14.0 25.0 14.0 16.0	[119]
*Vachellia nilotica* (L.) P.J.H.Hurter & Mabb. (*Acacia nilotica* Wild.)	Fabaceae	Leaves Fruits Twigs	*Chaetomium* sp. *Emericella* sp.1 *Alternaria* sp. *Mucor* sp. *Aspergillus* sp. *Chrysosporium* sp. *Curvularia* sp. *Emericella* sp. *Fusarium* sp. *Phoma* sp.	Methanol	Disc diffusion	8.0 7.3 22.7 10.3 7.3 9.3 20.6 7.7 NI NI	20.0 8.2 18.3 10.3 10.0 10.0 20.6 9.7 7.3 NI	[116]
*Ocimum basilicum* L.	Lamiaceae	Leaves Stem Flowers	*Cladosporium* sp. *Nigrospora* sp. *Curvularia* sp. *Chaetomium* sp. *Phoma* sp. *Aspergillus niger* *Alternaria* sp. *Geotrichum* sp.	Ethyl acetate Methanol	Cup diffusion	8.7–9.3 NI NI–11.7 NI–8.7 7.3–10.0 11.0–11.0 7.7–10.3 9.0–12.3	NI NI NI–8.0 NI–10.0 NI 7.7–9.7 NI–14.3 NI–7.0	[120]
*Cymbopogon schoenanthus* subsp. *proximus* (Hochst. ex A.Rich.) Maire & Weiller (*Cymbopogon proximus* (Hochst. ex A.Rich.) Stapf)	Poaceae	Leaves, Stem Bark	*Curvularia* sp. *Pleospora* sp.	Ethyl acetate	Disc diffusion	21.0 26.0	15.0 14.0	[119]

ZI: Zone of inhibition, *C. albicans*: *Candida albicans*, *A. niger*: *Aspergillus niger*, NI: No inhibition. Scientific names (in parentheses) are as mentioned by the author in the original publication.

## 6. Conclusions

Medicinal plants have emerged as important sources of antifungal agents, although many species of Sudanese medicinal plants have not been studied yet. In this review, a total of 88 plant species belonging to 51 families were investigated for antifungal activity, predominantly against species of *Candida*, *Saccharomyces* and *Aspergillus*. Among these, 16 species were specifically tested against *M. mycetomatis*. The family Fabaceae was the most screened family. Analysis of the publications reviewed indicated that the majority of papers used the cup-plate diffusion method for assaying antifungal activity. The MIC was the most prominent assay used to detect anti-*M. mycetomatis*. Our review particularly emphasizes that crude extracts from the Combretaceae family, *B. papyrifera* and *P. anisum* EO, and fungal endophytes (specifically *Alternaria*, *Curvularia*, and *Pleospora* spp.) isolated from *V. nilotica* and *Cymbopogon schoenanthus* exhibit significant antifungal activity. However, to translate these preliminary findings into therapeutic candidates, future research must address key scientific gaps rather than relying on routine screening. Priorities should focus on:

1. Bioassay-guided isolation and chemical characterization of pure bioactive metabolites from these high-priority species.
2. Evaluating mechanisms of action, systemic toxicity, and in vivo efficacy.
3. Testing synergistic combinations between plant/endophytic metabolites and conventional antifungal drugs to lower dosage and reduce resistance.
4. Transitioning to standardized high-throughput screening methods to ensure reproducibility across laboratories.
5. Bridging the gap in Sudanese endophytic microflora research to move beyond conventional plant tissue extraction and uncover novel natural products.

## Data Availability

No new data were created or analyzed in this study. Data sharing is not applicable to this article.

## Supplementary Materials

The following supporting information can be downloaded at: https://www.mdpi.com/article/10.3390/plants15203163/s1, Table S1: Methodological parameters for antifungal evaluations of Sudanese medicinal plants; Table S2: Methodological parameters for anti-fungal evaluations of endophytic fungi isolated from Sudanese medicinal plants.
