# Supplementary material for: Sudanese Medicinal Plants and Their Endophytic Fungi as Sources of Antifungal Agents
Source: Plants (Basel). 2026 Oct 15;15(20):3163. doi: 10.3390/plants15203163 (PMC13709147; doi:10.3390/plants15203163)
Supplement: Supplementary file 1 [file plants-15-03163-s001.zip › Table S1.pdf]

Table S1. Methodological parameters for antifungal evaluations of Sudanese medicinal plants.

Scientific names	Solvent control	Positive control (Concentration)/ ZI, mm	Number of replicates	Incubation condition	References
H. auriculata<br>*G. alata*<br>*R. indica*<br>*C. anthemoides*<br>*C. pratensis*<br>*R. indica*<br>*C. hartmannianum*<br>*E. racemosa* subsp. *Schimper*<br>*A. indica*<br>*L. inermis*<br>*A. pannosum*<br>*H. vitifolius*<br>*W. indica*<br>*C. cyphopetalum*<br>*C. quadrangularis*<br>*C. cyphopetalum*<br>*C. quadrangularis*<br>*H. thebaica*<br>*L. obtusifolium*	-	Nystain (12.5 μg/ml)/ 21, 23 mm<br>Clotrimazol (5 μg/ml)/ 15, 30 mm	-	-	[47]
E. longifolius<br>*A. nilotica*<br>*L. erecta*<br>*L. adscendens*<br>*P. irregularis*<br>*N. oleracea*<br>*N. pectinata*<br>*L. urticifolia*<br>*C. barbatus* var. *barbatus*<br>*T. purpurea*	-	Nystain (12.5 μg/ml)/ 21, 23 mm<br>Clotrimazol (5 μg/ml)/ 15, 30 mm	-	25°C for 7 days	[50]

Scientific names	Solvent control	Positive control (Concentration)/ ZI, mm	Number of replicates	Incubation condition	References
E. polyacantha<br>*J. aceroides*<br>*E. cuneata*<br>*E. scordiifolia*<br>*B. suffruticosa*<br>*C. pentagonum*<br>*T. leiocarpa*<br>*T. arjuna*<br>*C. demersum*<br>*G. lotoides*					
P. crispa<br>*P. undulata*	-	Clotrimazole / 26 mm<br>Nystatin / 14 mm	3	37°C for 48 h	[64]
G. alata	-	-	-	-	[72]
E. rauwolfii<br>*E. capensis*<br>*C. colocynthis*	-	-	-	-	[73]
S. birrea<br>*B. nigra*<br>*A. visnaga*	-	-	3	25°C for 48 h	[70]
B. papyrifera	-	-	3	25 °C for 5 days	[74]
H. thebaica	80% methanol<br>5% DMSO	Clotrimazole (5 mg/ml)/ 32-38 mm<br>-	-<br>-	35 °C for 48 h<br>25°C for 2 days	[53]<br>[71]
H. abyssinica	-<br>-	Nystatin (25 mg/ml)/ 19-18 mm	5<br>-	25°C for 10 - 14 days<br>25°C for 2 – 3 days	[49]<br>[50,51]
C. hartmannianum<br>*F. vasta*	-	-	-	25°C for 2 – 3 days	[51]
S. leptocarpa	-	-	-	25°C for 2 – 3 days	[51]

**Scientific names**	**Solvent control**	**Positive control (Concentration)/ ZI, mm**	**Number of replicates**	**Incubation condition**	**References**
C. sativa	-	Nystatin (50 µg/ml)/ 17, 28 mm<br>Clotrimazole (20 µg/ ml)/ 24, 43 mm	2	25°C for 1 day for the yeast and 3 - 5 days for *A. niger*	[47]
M. oblongifolia<br>*C. farinosa*<br>*S. nigrum* L.<br>*S. occidentalis*	Methanol	Nystatin (50 µg/ml)/ 17, 28 mm<br>Clotrimazole (20 µg/ ml)/ 24, 43 mm	3	-	[75]
M. oblongifolia	Methanol	-	3	-	[76]
T. brownii	Different negative control	Amphotericin B (0.030–1000 µg/mL)/ 7-26.33 mm; MIC 3.9–62.5 µg/mL	3	The 96-well microplates were incubated in an orbital shaker for 48 h, 200 RPM at 37 °C.; dishes were incubated at +35 °C for 48 h	[37]
C. africana	-	Clotrimazole (40 µg/ml)/ 30, 42 mm<br>Nystatin (50 µg/ml)/ 28, 17 mm	2	25°C for 2 days	[77]
C. amarus	-	-	3	37°C for 48 h	[78]
C. maxima	-	Clotrimazole (40 µg/ml)/ 42-32 mm<br>Nystatin (40 µg/ml)/ 32-27 mm	-	25°C for 2 days for the *C. albicans* and 3 days for *A. niger*	[79]
C. rotundus	-	Clotrimazole (40 µg/ml)/ 30-42 mm<br>Nystatin (50 µg/ml)/ 28-17 mm	-	25°C for 2 days for *C. albicans* and 3 days for *A. niger*	[80]
H. sabdariffa<br><br>*R. communis*	-	Clotrimazole (20 mg/ml)/ 34-24 mm<br>Nystatin (25 mg/ml)/ 26-14 mm	2	-	[81]
A. hamiltoniana<br><br>*V. seyal* var. *fistula*	-	Clotrimazole (30 mg/ml)/ 38 mm	3	25°C for 4 days	[65]

Scientific names	Solvent control	Positive control (Concentration)/ ZI, mm	Number of replicates	Incubation condition	References
S. polyacantha					
V. nilotica	-	Clotrimazole (40 µg/ml)/ 30-42 mm<br>Nystatin (50 µg/ml)/ 28-17 mm	2	25°C for 2 days for the *C. albicans* and 3 days for *A. niger*	[82]
T. foenum-graecum	%10 DMSO	-	3	25°C for 2 days for the *C. albicans* and 3 days for *A. niger*	[68]
D. cinerea	-	-	-	-	[83]
P. acaciae	-	Clotrimazole (20 µg/ml)/ 21 mm<br>Nystatin (50 µg/ml)/ 26 mm	4	-	[84]
L. inermis	-	Nystatin (25 µg/ml)/ 17.75-16.00 mm	3	25°C for 10 - 14 days	[48]
A. digitata<br>*Z. spina-christi*<br>*H. sabdariffa*	5% DMSO	-	-	25°C for 2 days	[71]
A. digitata	-	Fluconazole/ 27, 26 mm	-	-	[52]
	-	Clotrimazole (40 µg/ml)/ 42-32 mm<br>Nystatin (40 µg/ml)/ 32-27 mm		25°C for 2 days for the *C. albicans* and 3 days for *A. niger*	[79]
	-	Clotrimazole (40 mg/ml)/ 28 mm	-	25°C for 4 days	[69]
C. tridens	-	Nystatin (10 µg/disc)/ 22, 20 mm	-	-	[85]
H. sabdariffa	30% DMSO	-	-	-	[86]
A. indica	-	-	2	25°C for 72 h	[66]
M. oleifera	-	-	2	25°C for 10 - 14 days	[87]
X. americana	-	Clotrimazole (5 µg/ml)/ 16,30, 15 mm<br>Nystatin (10 µg/disc)/ 14, 26, 24 mm	-	-	[88]
P. oleracea	100% DMSO	Nystatin (10 µg/disc)/ 22, 20 mm	-	25°C for 2 - 3 days	[46]

**Scientific names**	**Solvent control**	**Positive control (Concentration)/ ZI, mm**	**Number of replicates**	**Incubation condition**	**References**
N. sativa	-	Clotrimazole (40 μg/ml)/ 30-42 mm<br>Nystatin (50 μg/ml)/ 28-17 mm	2	25°C for 2 days for *C. albicans* and 3 days for *A. niger*.	[89]
Z. spin- christi	-	-	2	25°C for 3 days for the *A. niger* and 2 days for *C. albicans*.	[90]
D. stramonium	Petroleum ether<br>Methanol	-	-	-	[91]
C. longa	-	-	-	-	[92]

DMSO: Dimethyl sulphoxide; ZI: Zone of inhibition; -: Not available.
