# Supplementary material for: Sudanese Medicinal Plants and Their Endophytic Fungi as Sources of Antifungal Agents
Source: Plants (Basel). 2026 Oct 15;15(20):3163. doi: 10.3390/plants15203163 (PMC13709147; doi:10.3390/plants15203163)
Supplement: Supplementary file 1 [file plants-15-03163-s001.zip › Table S2.pdf]

Table S2. Methodological parameters for antifungal evaluations of endophytic fungi isolated from Sudanese medicinal plants.

**Scientific name**	**Solvent control**	**Positive control (Concentration)/ ZI, mm**	**Number of replicates**	**Incubation condition**	**References**
K. africana	-	Antifungal standard (without mention the name)	2	-	[119]
C. schoenanthus					
V. nilotica	-	Nystatin(10/µg/disc)/ 16, 30 mm	-	-	[116]
O. basilicum	DMSO	Nystatin (100,000 IU/ml)/ 21, 29 mm	-	At room temperature for 24 h for *C. albicans* and 48 h for *A. niger*	[120]

DMSO: Dimethyl sulphoxide; ZI: Zone of inhibition; -: Not available.
